# To save or not to save your family member’s life? Evolutionary stability of self-sacrificing life history strategy in monogamous sexual populations

**DOI:** 10.1186/s12862-019-1478-0

**Published:** 2019-07-19

**Authors:** József Garay, Barnabás M. Garay, Zoltán Varga, Villő Csiszár, Tamás F. Móri

**Affiliations:** 1 MTA Centre for Ecological Research, Evolutionary Systems Research Group, Klebelsberg Kuno u. 3, Tihany, H-8237 Hungary; 2 MTA-ELTE Research Group in Theoretical Biology and Evolutionary Ecology and Department of Plant Systematics, Ecology and Theoretical Biology, ELTE Eötvös Loránd University, Pázmány P. s. 1/C, Budapest, H-1117 Hungary; 3 Faculty of Information Technology and Bionics, Pázmány Péter Catholic University, Práter u. 50/A, Budapest, H-1083 Hungary; 4 Department of Mathematics, Szent István University, Páter K. u. 1, Gödöllő, H-2103 Hungary; 5 Department of Probability Theory and Statistics, Eötvös Loránd University, Pázmány P. s. 1/C, Budapest, H-1117 Hungary

**Keywords:** Altruism, Hamilton’s rule, Mating table, Morality, Monogamy

## Abstract

**Background:**

For the understanding of human nature, the evolutionary roots of human moral behaviour are a key precondition. Our question is as follows: Can the altruistic moral rule “Risk your life to save your family members, if you want them to save your life” be evolutionary stable? There are three research approaches to investigate this problem: kin selection, group selection and population genetics modelling. The present study is strictly based on the last approach.

**Results:**

We consider monogamous and exogamous families, where at an autosomal locus, dominant-recessive alleles determine the phenotypes in a sexual population. Since all individuals’ survival rate is determined by their altruistic family members, we introduce a new population genetics model based on the mating table approach and adapt the verbal definition of evolutionary stability to genotypes. In general, when the resident is recessive, a homozygote is an evolutionarily stable genotype (ESG), if the number of survivors of the resident genotype of the resident homozygote family is greater than that of non-resident heterozygote survivors of the family of the resident homozygote and mutant heterozygote genotypes. Using the introduced genotype dynamics we proved that in the recessive case ESG implies local stability of the altruistic genotype. We apply our general ESG conditions for self-sacrificing life history strategy when the number of new-born offspring does not depend on interactions within the family and the interactions are additive. We find that in this case our ESG conditions give back Hamilton’s rule for evolutionary stability of the self-sacrificing life history strategy.

**Conclusions:**

In spite of the fact that the kidney transplantations was not a selection factor during the earlier human evolution, nowadays “self-sacrificing” can be observed in the live donor kidney transplantations, when the donor is one of the family members. It seems that selection for self-sacrificing in family produced an innate moral tendency in modulating social cognition in human brain.

**Electronic supplementary material:**

The online version of this article (10.1186/s12862-019-1478-0) contains supplementary material, which is available to authorized users.

## Background

For the understanding of human nature, the evolutionary roots of human moral behaviour are a key precondition. The self-sacrificing behaviour within family is a social norm in most cultures. In the paper we apply the basic idea of evolution stability for our new population genetics model to investigate when the self-sacrificing behaviour within family is evolutionary rational. In evolution, from eusociality [1], through parental care [2] to sib cannibalism [3], interaction occurs between family members, i.e. between parents and their offspring and between siblings. For modelling this selection situation, there are basically three different approaches, all of which are connected to the present paper.

The first one uses the classical evolutionary game theory model [4, 5], based on the fact that interaction takes place between two individuals and the pay-off depends on the strategies of the players. For instance, if cooperation is evolutionarily stable in a well-mixed population [6, 7], then it should also be stable among siblings. However, classical evolutionary game theory is based on the following two assumptions, neither of which hold in our case. The first assumption is that the interaction is well-mixed (i.e. the rate of interaction between different phenotypes is proportional to their relative frequency in the whole population). Clearly, interaction within the family is not well-mixed. On the one hand, it has been pointed out [8] that if the interaction rate is high enough within the same asexual phenotype, then the maximizer phenotype (maximizing the average fitness coming from the interactions within the same phenotype) overperforms the classical evolutionarily stable strategy, ESS. (We note that there are a number of evolutionary studies on non-well-mixed interactions [9–12]. Moreover, our problem is connected to *n*-person games [12, 13], since the family can be considered as a genetically well-defined group. The second basic assumption of classical evolutionary game theory is that the population is asexual. However, parental care occurs in sexual families. We note that the relationship between the predictions of asexual and sexual evolutionary models is not a straightforward issue [14, 15].

The second approach uses kin selection theory. Hamilton [16] considered interactions between “neighbours” in a non-overlapping sexual viscous population, he measured the average genetic relatedness between neighbours and used a gene pool approach. Moreover, in this model interactions take place between two individuals. Under these assumptions, Hamilton’s rule claims that the frequency of altruistic genes should increase in a sexual population if *rB* > *C*, where *r* is the genetic relatedness of the recipient to the actor, *B* is the additional reproductive benefit gained by the recipient of the altruistic act and *C* is the reproductive cost to the individual performing the act. Observe that Hamilton’s rule applies to an interaction between recipient and actor, and only focuses on the degree of their genetic relatedness, furthermore it does not take into account the genotype-phenotype mapping. We note that Hamilton’s rule was validated by Rowthorn [17] in a sexual model where two alleles code different levels of altruism. Moreover, van Veelen [18] found that the mean inclusive fitness is maximized if there is no pair of alleles for which there is over- or underdominance. We also note that in a sexual model for parent-offspring conflict [19, 20], Hamilton’s rule could not be validated by Bossan et al. [21].

We call the attention to the fact that now we will strictly focus only on the genes that determine the altruistic interactions within a monogamous family, disregarding the competition between families. In this case, the distribution of altruistic genes in groups of families has no direct effect on the evolutionary success of the genes considered within the family. Thus our model cannot be built upon any of the earlier life history models [22, 23], or Price’s equation [24]. We note that the models based on Price’s equation support Hamilton’s rule [25, 26].

Before describing the third modelling approach and setting up ours, we recall some recent results we consider starting points of the present study. In Garay et al. [3] a kin demographic selection model was introduced for the study of the selection situations when interactions take place only within a family of an asexual aging population with overlapping generations. Here fitness was the long-term growth rate of the phenotype, and it was shown that cannibalism can be considered as a mutualistic kin strategy, if the sib cannibal either decreases its developmental time or increases fecundity in the adult stage. There are two further applications of this kin demographic selection model. In Garay et al. [27] the juvenile honest food solicitation and parental investment was studied. It was shown that honest begging results in decreased variance of collected food among siblings, which leads to a higher mean number of surviving offspring. Furthermore, in Garay et al. [28] the evolutionary roots of human morality were studied. It was found that the biological version of the Fifth commandment, called the Fifth rule (“*During your reproductive period, give away from your resources to your post-fertile parents*”) can spread by means of natural selection, by increasing the survival rates of the family members. All three studies cited above considered asexual populations, thus the question arises: What is the mathematical condition for the evolutionary stability of altruistic behaviour within the monogamous family in a diploid sexual population?

To answer the latter question, we will follow the third modelling approach, namely we will use a population genetic model that can take account of the genetic background of the inheritance of the altruistic phenotype, and the frequency dependent interactions between genotypes, at the same time [29–31]. The advantages of a population genetic model for the evolution of altruism were already demonstrated back in the 1980’s, [32–34]. Maynard Smith [30] has already pointed out that the personal fitness model has the virtue of “*incorporating the evolution of altruism into the corpus of population genetics as an example of frequency-dependent selection*.” It seems to us that this research line, in comparison with the kin and group selection approaches, is less elaborated. The reason for this, as we see it, is that the study of population genetics models may be mathematically rather involved, although recently these models gained more attention [17, 35]. Furthermore, based on Hamilton [36] Michod [37, 38] pointed out that inbreeding (e.g. sib mating) may facilitate the evolution of altruism [39]. However, the incest has been one of the most widespread of all cultural taboos, both in present and in many past societies [40]. What is more, in Israeli kibbutzim, it was observed that there was no sexual activity and no marriage between those who are not relatives but grew up in the same peer group. Among marriages of second generation adults in all kibbutzim, no intra-peer group marriage occurred; in fact, a continuous exposure among peers aged 0–6 years results in sexual avoidance and exogamy [41]. Based on all these, here we only focus on the exogamous, monogamous families (in particular, we will not consider either polygamy, or sexual selection). We note that, for the sake of simplicity, we do not investigate either the stability of monogamy [42], or the effect of the cultural evolution [43]. The reason for the latter is our hypothesis that the evolutionary roots of the moral may originate from the times before the evolution of language. Since we are strictly interested only in what happens within monogamous families, our population genetics model is rooted in the models of Hull [44] and Haldane and Jayakar [45], where the juveniles’ survival rate depends on the genotypes of their parents. The basic assumptions of the here introduced population genetics model are quite different from those of Hamilton [16, 32, 37], and are as follows: 1. We consider a large enough, non-ageing, sexual population with overlapping generations [17, 21]. 2. The mating system is exogamous and monogamous [46] without promiscuity and in each reproductive season the pair formation is random. 3. Interactions take place only within each family, and they determine the survival rate of the family members. Thus we consider the following population cycle: Firstly, the pairs of parents are formed randomly, so the mating system is not viscous. Secondly, the family has offspring, whose phenotype is determined by the genotypes of the parents according to Mendelian inheritance. Thirdly, the survival rate of each family member is determined by the phenotypes of family members. Then the cycle starts again. In spite of the fact that the random mating happens between zygotes in the whole population, in our phenotypic selection situation each individual’s survival rate is determined by her family members, and the genotype composition of each family depends on the genotypes of monogamous pairs of parents, thus our selection situation motivates us to use the mating table approach as a starting component of the population genetics model introduced above. A complete dynamical characterization of genotype distribution changes is not our goal. We are interested only in the evolutionary stability of a genotype distribution in a sexual population: Under what conditions is a homozygote state stable?

**Fig. 1:**
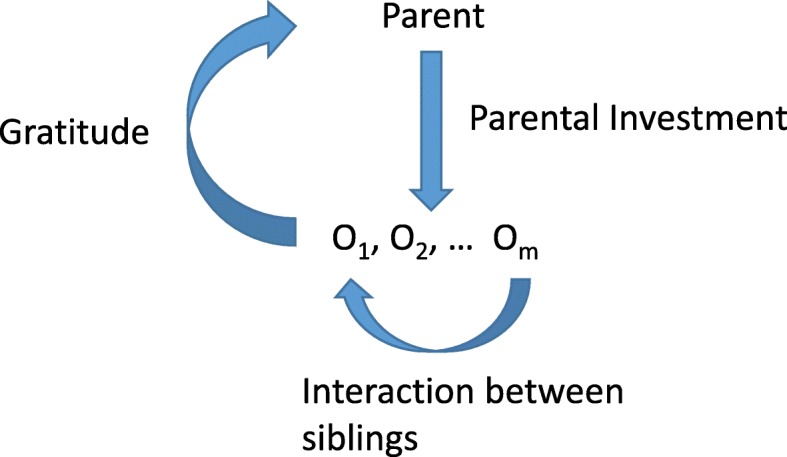
The arrows visualize interactions, + and –stands for gain and cost, respectively

In the present paper we focus on the following “self-sacrificing” norm: “*Risk your life to save your family members, if you want them to save your life*.” In biological terms, this norm of self-sacrificing means that the actors risk their own lives for the lives of their family members, i.e. the altruistic interactions change the survival rate of the family members. (In Additional file 1: SI A, we give a fairly general method for the biological modelling of a moral norm.) We note that the nomenclature of “self-sacrificing” goes back to the Haldane quip [47]: “*Legend has it that in a pub one evening Haldane told his friends that he would jump into a river and risk his life to save two brothers, but not one, and that he would jump in to save eight cousins, but not seven*.” We consider a monogamous family in which male and female differ only in sex, i.e. they are the same from all other points of view. Furthermore, we consider three interactions within the family (see in Fig. 1).

The first interaction is parental investment, i.e. when parents, at the expense of decreasing their own survival rate, increase the survival rate of their offspring. We note that, in human, birth is risky, in our terminology self-sacrificing, e.g. in 2015 the global maternal mortality ratio was 216 deaths per 100 000 live births [48]. For simplicity, we consider two levels of parental investment: a *provider parent* invests more than a *non*-*provider parent*. The second interaction is *sib altruism*, i.e. the altruistic juvenile individuals, at the expense of decreasing their own survival rate, increase the survival rate of their siblings, while the *non-altruistic siblings* do not help others. The third interaction is *offspring gratitude,* i.e. juvenile individuals, at the expense of decreasing their own survival rate increase the survival rate of their parents, while non-grateful siblings do not. We remind that from psychological point of view, human gratitude appears during childhood [49–51]. We use the term “offspring gratitude” to emphasize the difference from other kinds of gratitude as e.g. “upstream reciprocity” [52, 53], and the Fifth rule [28]. Now we can make precise what we understand under “*self-sacrificing life history strategy*”: an individual having this phenotype is altruistic and grateful in juvenile age, and is a provider parent during adult age. Our question can now be formulated as follows: Under what conditions will the self-sacrificing life history strategy be evolutionary stable in sexual populations, if mutation is rare enough?

In this paper we introduce a new population genetics model, with three main simplifying assumptions: during pre-human and early human evolution there was no family planning, the offspring numbers are fixed (i.e. the offspring number in families does not depend on the interactions within the family), and the populations are non-aging. We conclude our study with a discussion section.

## Results

First we give the details of the genetic system we will use: We consider a population of (large) size *N* with sex ratio half-half, with no sexual selection, i.e. the females and males are different only in their sex.

### Mating system

We consider well-mixed reproductive pair formation between genotypes in the whole population. The species is monogamous with internal fertilization, so *N*/2 couples are formed at random, and each couple breeds a fixed number *n* of offspring. Thus the probability of two genotypes’ mating depends only on their frequencies in the population, so there is no assortative mating. Thus, the degree of relatedness between full siblings is ½. We emphasize that if the population is large enough, then the random mating excludes inbreeding.

### Genetic composition

We consider a three-dimensional life history strategy. For simplicity, we assume each of them is coded by only one autosomal locus, exactly two alleles. Assume these loci are located on the same autosomal chromosome. (Since later we assume that mutation is rare, if the three loci are on different chromosomes, our calculation still applies.)

### Mendelian genotype-phenotype map

We denote the altruistic/non-altruistic alleles by a/A, the grateful/non-grateful alleles by b/B, and the provider/non-provider ones by c/C. We will study two cases: when the self-sacrificing alleles are recessive, and when they are dominant with respect to the mutant alleles [30, 32]. Let *K* be the number of genotypes in the population, denote the genotypes by G_*i*_, *i =* 1, 2, …, *K.* In theory, *K* = 27 genotypes are possible (three possibilities on each locus), but since we consider rare mutations, *K* will be a smaller number. Our *main assumption* on the genotype-phenotype map is the following: In the resident sexual population, each individual has self-sacrificing phenotype, coded by a totally homozygote genotype. This kind of realization is a prerequisite for the sexual population to evolve to the optimal state of the asexual population [54].

### Interaction within the family

Clearly, the parents’ genotypes determine the behaviour of the parents and the genotype distribution of theirs offspring, thus the behaviour of the offspring as well. Consequently we can use the following notation: If the parents’ genotypes are G_*i*_ and G_*j*_, let the parents’ survival probability be *q*_*i*(*ij*)_ and *q*_*j*(*ij*)_, respectively, and let the average number of survived offspring with genotype G_*k*_ be *n*_*k*(*ij*)_ (cf. [44, 45]). Observe that our model is general in the sense that *n*_*k*(*ij*)_ can be given by an evolutionary game with arbitrary many players.

#### General conditions for the evolutionary stability of genotypes

Now we ask when the self-sacrificing genotype is an *evolutionarily stable genotype* (ESG) in the arbitrarily large sexual population. Following Maynard Smith and Price [5], we say a stage is ESG if the rare mutant gene cannot invade into the monomorphic resident population. Mathematically this means that the (relative) frequency of the resident genotype increases from generation to generation, provided mutation is sufficiently rare. If mutation is very rare, there is time enough for the fixation of the best genotype between two mutations. On the other hand, if mutation is rare enough, only one type of mutation occurs at a time in the resident monomorphic [a,b,c] population, i.e. only one of [a,b,c] → [A,b,c], [a,b,c] → [a,B,c] and [a,b,c] → [a,b,C] mutation appears. Our assumption on mutation is parallel with the streetcar theory by Hammerstein [55], since if mutation is very rare, then the [A,B,c] chromosome can appear only if either a → A mutation occurs first, and after the fixation of the A allele, b → B mutation occurs, or conversely, etc. Finally, since mutation is very rare, the relative frequency of the mutant is very small.

Now consider a mutation, e.g. a → A. We have the following diploid genotypes: G_1_ = ([a,b,c],[a,b,c]), G_2_ = ([a,b,c],[A,b,c]) and G_3_ = ([A,b,c],[A,b,c]), with relative frequencies *y*_1_, *y*_2_, and *y*_3_, respectively, which compose the frequency vector *y* = (*y*_1_, *y*_2_, *y*_3_). The genotype survival table based on the mating table is as follows (see Table 1):

**Table 1:** Genotype survival table based on the mating table

Genotypes of parents	Average number of couples	Probability distribution of offspring, and Average number of survived family members, for genotypes G_1_, G_2_, G_3_		
		G_1_	G_2_	G_3_
G_1_xG_1_	$\frac{Ny_1^2}{2}$	1	0	0
		2*q*_1(11)_ + *n*_1(11)_	0	0
G_1_xG_2_	*Ny*_1_ *y*_2_	½	½	0
		*q*_1(12)_ + *n*_1(12)_	*q*_2(12)_ + *n*_2(12)_	0
G_1_xG_3_	*Ny*_1_ *y*_3_	0	1	0
		*q*_1(13)_	*n*_2(13)_	*q*_3(13)_
G_2_xG_2_	$\frac{Ny_2^2}{2}$	¼	½	¼
		*n*_1(22)_	2*q*_2(22)_ + *n*_2(22)_	*n*_3(22)_
G_2_xG_3_	*Ny*_2_ *y*_3_	0	½	½
		0	*q*_2(23)_ + *n*_2(23)_	*q*_3(23)_ + *n*_3(23)_
G_3_xG_3_	$\frac{Ny_3^2}{2}$	0	0	1
		0	0	2*q*_3(33)_ + *n*_3(33)_

For instance, consider a family of parents with G_1_ and G_2_ genotypes. This family has G_1_ offspring with probability ½, and G_2_ offspring with probability ½, as well. Thus the genotype distribution in this family is given by a binomial distribution, i.e. $$ \left(\begin{array}{c}n\\ {}k\end{array}\right){\left(\frac{1}{2}\right)}^n $$ is the probability that this family has exactly *k* offspring with genotype G_1_. The same table applies for the two other mutations b → B and c → C.

The difficulty in modelling this process is that the allele distribution cannot determine the genotype distribution, because random mating happens between zygotes and not between gametes (in our case, the fertilisation is internal and there is no promiscuity), and the selection depends on the genotypes of monogamous pair of parents [44, 45]. Consequently, the states of our system must be the parent genotype distributions, since in the next generation the allele distribution cannot give the genotype distribution as in the case when the gametes mate randomly. For instance, in the next generation the relative frequency of a heterozygote genotype is not equal to the double product of the relative frequencies of the alleles in the heterozygote genotype. In other words, in our model the genotype is the unit of the evolution (i.e. the replicator).

As we already mentioned, we adapt the intuitive definition of evolutionary stability by Maynard Smith and Price [5]: A state is evolutionary stable, if the rare mutant cannot invade into the monomorphic resident population. In other words, if the growth rate of genotype G_1_ is higher than the average growth rate of the whole population, then a rare enough mutant dies out. This paradigm idea is formalized in SI. B. There we investigate necessary and sufficient conditions for the evolutionary stability of a genotype G_*i*_ in a general setting. In our special case, we only have three genotypes, G_1_ is the resident genotype, G_2_ is a primary mutant genotype, and G_3_ is a secondary mutant. Primary mutants are ones which can appear spontaneously at a certain time, while secondary mutants can only be born to mutant-mutant couples. Hence, if the frequency of primary mutants is of order ε, then the frequency of secondary mutants is only of order ε^2^. In SI (see condition (ii) of Theorem 1 in SI B), we show that in this setting, a simple sufficient condition for the evolutionary stability of the resident genotype is the following:

*First order condition*:

1 $$ 2{q}_{1(11)}+{n}_{1(11)}>2\left({q}_{2(12)}+{n}_{2(12)}\right).\kern0.75em $$

This means that the growth rate $\frac{2{q}_{1(11)}+{n}_{1(11)}}{2}$ of the resident G_1_xG_1_ family has to be larger than the growth rate $\frac{q_{2(12)}+{n}_{2(12)}}{1}$ of genotype G_2_ in a G_1_xG_2_ family formed by resident and mutant heterozygote parents. Observe that Hamilton’s rule (concerning pair of actors) and the first order condition (1) are different.

Heuristically, the reason behind condition (1) is as follows. Initially, the number of G_1_ individuals in the population is approximately *N* (plus a term of order ε). In the next generation, the number of G_1_ individuals is about $\frac{N}{2}\left(2{q}_{1(11)}+{n}_{1(11)}\right)$, plus terms of smaller order. Therefore, the growth rate of this genotype, as the mutation frequency ε tends to zero, is $\frac{2{q}_{1(11)}+{n}_{1(11)}}{2}$. On the other hand, the number of G_2_ individuals in the initial population is approximately *Nε* (plus a term of order ε^2^). In the next generation, the number of G_2_ individuals is *Nε*(*q*_2(12)_ + *n*_2(12)_), plus terms of smaller order. Therefore, the growth rate of this genotype, as the mutation frequency ε tends to zero, is *q*_2(12)_ + *n*_2(12)_. Genotype G_1_ is ESG, if it has a higher growth rate than genotype G_2_.

Observe that if the mutant allele A is recessive under allele a, then genotypes G_1_ and G_2_ have the same phenotype, thus *q*_1(11)_ = *q*_1(12)_ = *q*_2(12)_ and *n*_1(11)_ = 2*n*_1(12)_ = 2*n*_2(12)_. Thus in condition (1) above, equality holds. In other words, when the resident allele is dominant over the mutant one, then with respect to condition (1), the mutant is neutral in the sense that the most abundant families G_1_G_1_ and G_1_G_2_ are phenotypically the same. Condition (iii) of Theorem 1 in SI B gives a sufficient *second-order condition* for genotype G_1_ to be an ESG in this case as well. In these conditions the other, less frequent parent couples also appear (e.g. mating G_2_xG_2_). This shows that the genotype-phenotype mapping can change the conditions of the ESG.

Evolution is a dynamical process, and the notion of evolutionary stability relies upon an implicit dynamical consideration, such as the classical matrix game and replicator dynamics [56]. In SI C we have introduced a genotype dynamics for the frequency vector of the genotypes. In a general setting, in Theorem 2 we have proved that the resident population containing recessive homozygotes, i.e. *y*^∗^ = (1, 0, 0), is locally asymptotically stable if in a neighbourhood of *y*^∗^ = (1, 0, 0), the growth rate of genotype G_1_ is higher than the average growth rate of the whole population.

#### Application: conditions for the evolutionary stability of the self-sacrificing phenotype in the additive model

In this section we apply our general result for the simplest, additive situation, since additivity gives a chance to make clear the connection between Hamilton’s rule and our ESG conditions [57], see SI B. We consider the special case when the costs and benefits of altruism and gratitude are additive. We denote by *b* and *c*_*a*_ the benefit and cost of altruism, respectively. Also, *γ* and *c*_*g*_ denote the benefit and cost of gratitude, respectively, where we suppose that each offspring helps *only one of its parents, chosen randomly*. (We note that if each offspring helps both parents, then the individual cost of gratitude is 2*c*_*g*_ and the benefit of each parent is *γ*. For this case we get the same condition.) Regarding parental investment, the baseline survival probability of a parent with investment *F*_*i*_ is still *q*_*i*_ (with *q*_1_ **<** *q*_2_), but we now have three types of families according to the number of provider parents. We denote by *π*_*i*_ the baseline survival probability of an offspring with *i* provider parents, *i* = 0, 1, 2. Clearly, *π*_2_ > *π*_1_ > *π*_0_.

Let us find conditions under which the self-sacrificing genotype is an ESG. In the next subsections, we show the calculations for the case of dominant mutation, while for recessive mutation, we only give the results, and refer to SI B for the derivation.

Introduce *η* as the number of G_2_ offspring born in a G_1_xG_2_ family, which has a binomial distribution with parameters *n* and ½.

### Non-altruistic mutant

Since altruism among siblings does not affect parents’ survival rates, *q*_1(11)_ = *q*_2(12)_. Hence, condition (1) simplifies to *n*_1(11)_ > *n*_2(12)_. If the non-altruistic mutation is dominant, then

$$ {n}_{2(12)}=\frac{1}{2}{n}_{1(11)}+E\left(\eta \left(\eta -1\right){c}_a-\eta \left(\eta -1\right)b\right)=\frac{1}{2}{n}_{1(11)}+\frac{1}{2}n\left(n-1\right){c}_a-\frac{1}{4}n\left(n-1\right)b, $$

thus condition (1) is equivalent to

$$ b>2{c}_a. $$

It is shown in SI B.2.2 that this condition is necessary and sufficient for the altruistic allele to be an ESG. Also, we derive in SI B that if mutation is recessive, then *b* > 2*c*_*a*_ is a sufficient condition, while *b* ≥ 2*c*_*a*_ is necessary.

### Non-grateful mutant

Since in a G_1_xG_2_ family, each grateful offspring adds an average $\frac{\gamma }{2}$ to the G_2_ parent’s survival probability, we have

$$ {q}_{2(12)}={q}_{1(11)}-E\left(\eta \frac{\gamma }{2}\right)={q}_{1(11)}-\frac{1}{4} n\gamma . $$

Furthermore,

$$ {n}_{2(12)}=\frac{1}{2}{n}_{1(11)}+E\left(\eta {c}_g\right)=\frac{1}{2}{n}_{1(11)}+\frac{1}{2}{nc}_g. $$

Thus

$$ 2\left({q}_{2(12)}+{n}_{2(12)}\right)=2{q}_{1(11)}+{n}_{1(11)}-\frac{1}{2} n\gamma +{nc}_g, $$

Hence condition (1) is equivalent to

$$ \gamma >2{c}_g. $$

Similarly to the previous case, it is shown in SI B.2.1 that this condition is necessary and sufficient for the grateful allele to be ESG. Also, we derive in SI B.1.1 that if mutation is recessive, then *γ* > 2*c*_*g*_ is a sufficient condition, while *γ* ≥ 2*c*_*g*_ is necessary.

### Non-provider mutant

Now *n*_1(11)_ > *nπ*_2_, ${n}_{2(12)}>\frac{1}{2}n{\pi}_1$, *q*_1(11)_ > *q*_1_ and *q*_2(12)_ > *q*_2_. Hence condition (1) for the provider behaviour to be ESG is

$$ n\left({\pi}_2-{\pi}_1\right)>2\left({q}_2-{q}_1\right). $$

In SI B.2.3 it is derived that if equality holds in condition (1), then the second order condition is ${\pi}_1>\frac{\pi_0+{\pi}_2}{2}$, in other words, the benefit of two provider parents has to be less than the double of the benefit of a single provider. In the recessive case, *n*(*π*_2_ − *π*_1_) > 2(*q*_2_ − *q*_1_), is again sufficient for the provider allele to be an ESG.

## Discussion

We consider sexual models with the following assumptions: the population is overlapping and non-ageing, moreover, mutation is rare enough. We emphasize that our simplifying assumption, namely that the interactions within the family have no effect on the offspring number of the family, is not used in the general model, but is used in the applications.

In our study we strictly follow the Darwinian tenet: a genotype with higher growth rate than the average growth rate of the whole population will be fixed by the natural selection. In other words, we consider neither the inclusive fitness [58], nor the “welfare of family” (similarly to “welfare of group” [59]), as an objective function. We think that our model takes account of some basic ideas of the above two research lines, in this sense it is a partial combination of both approaches. The basic idea of the inclusive fitness theory is that genetic relatedness is important for the social evolution [60], and the mating table method gives a detailed description of the genetic relatedness. The basic idea of group selection theory is that the multi-level selection is important for social evolution. In the present paper, the altruistic interactions within the family determine the individual survival rate of all family members. However, the family has genetically related members, and our present model does not take into account the competition between families [61] and group-level phenomena (e.g. competition between groups formed by unrelated families, moreover fission, fusion, and extinction of groups [62]). Based on these, we hope that our model is a step towards a further population genetics model, which simultaneously takes account of the details of the genetic system (e.g. mating system, genotype-phenotype mapping etc.) and the details of multi-level selection. We feel that such type of further population genetics models could bring together the above basic ideas of the kin and the group selection theories [29–31], under the umbrella of the “orthodox” Darwinian view [63, 64].

### Evolutionary stability when interaction takes place within the family

We adapted the intuitive definition of ESS by Maynard Smith and Price to our population genetics model, and depending on whether the resident is dominant or recessive, we found the following two conditions for the ESG: When the resident is recessive under the mutant, then the number of survivors of the resident genotype (G_1_) in the resident homozygote family (G_1_G_1_) is greater than twice that of the non-resident heterozygote (G_2_) survivors in the family of the resident homozygote and heterozygote genotypes (G_1_G_2_). When the resident is dominant over the mutant, then the number of survivors of the resident genotype (G_1_) in the families of resident homozygote (G_1_G_1_) and the genotype contains mutant gene (G_1_G_2_ and G_1_G_3_), is greater than the number of non-resident heterozygote (G_2_), and mutant homozygote (G_3_) survivors in the families do not contain the resident homozygote (G_2_G_2_, G_2_G_3_ and G_3_G_3_). Observe that the conditions of evolutionary stability in the sexual model are sensitive to the genotype-phenotype mapping (see Theorem 1 in SI B), since when the resident is dominant over the mutant, phenotypically there is no difference between the resident homozygote family (i.e. G_1_G_1_) and the family of the resident homozygote and heterozygote genotype (i.e. G_1_G_2_) [32].

The models of Hamilton [16] and ours are quite different, since they are based on the gene pool and the mating table approaches, respectively. Consequently, the general conditions of the inclusive fitness approach and those of our evolutionary stability approach are quite different: The conditions of the first one are based on interactions between two individuals. Our ESG conditions, instead, are based on the different numbers of survivors of different genotypes in different families. In summary, our general conditions are not the same as Hamilton’s rule. However, in the additive model our first and second conditions (see conditions (ii) and (iii) of Theorem 1 in SI) are equivalent with Hamilton’s rule.

### Further possible biological applications

Although the present paper was motivated by a human moral rule, our model strictly belongs to the theory of evolution, and can be used when a group contains only family members. The introduced ESG conditions are general, thus they can be also used to other types of interactions within the family. For instance, if the resource is scarce, then e.g. a symmetric dove-hawk game can describe the interaction between siblings. Moreover, the interaction between parents and offspring can be given by Trivers’ model [19] etc. In other words, if the interactions between family members can be given by some reasonable *n*-person game, then the mean number of the survived members of different families can be calculated. Furthermore, the eusocial animals (e.g. naked mole-rat), certain mammals that exhibit some eusocial tendencies (e.g. meerkats, dwarf mongooses) and wolf, also exhibit offspring gratitude defined above. In the future our model can be extended to the case when sib altruism can increase the number of offspring in the family.

### Evolutionary root of morality

In the Introduction we focused on a partial version of golden rule: “*Risk your life to save your family members, if you want them to save your life.”* In biological terms, this rule can be described as self-sacrificing life history strategy. We have given sufficient conditions for the evolutionary stability of this strategy by pure biological reasoning. Thus we can say that this moral rule has an evolutionary root, in other words, this moral rule is evolutionarily rational. The above conditions for evolutionary stability of self-sacrificing life history strategy for the monogamous sexual model are valid, if all the considered different interactions within the family are efficient enough. This holds under such ecological condition that the self-sacrificing family can either effectively defend against predators, or successfully exploit the food resources, or both. The latter condition should have held during the evolution of our ancestors. Altruism can appear in a cooperation, e.g. when a predator attacks a family member, then other family members can help, and/or they cooperatively defend against predator [8, 65].

Obviously, all assumptions of our biological model do not hold for humans nowadays (e.g. the number is offspring is mostly not biologically, but rather socially determined). Nevertheless, the existence of altruism in human families is documented at present, e.g. the altruism is an important factor in family firms in USA [66]. Furthermore, “self-sacrificing” can be observed in kidney transplantations, when the live donor is one of the family members [67]. The estimated rate of transplants relying on live donations from family members was 80% in Mexico [68], 60% in USA [69], 37% in Taiwan, 99% in Japan, and 66% in South Korea [70].

Now the following question arises: Is there any evidence that the moral rule considered here has an innate tendency? We think that such evidence must be connected to the human brain, since it has been previously shown that differences in brain activity in different brain regions are associated with moral and emotional conflict regulation [71], decision making [72], mentalizing [73] and perspective-taking [74]. Bacha-Trams et al. [75] focused on the moral dilemma of Anna to donate one of her kidneys to her sister Kate, who is fatally ill with cancer. One group of subjects were told that the sisters were genetic sisters, the other group was told that Anna had been adopted as a newborn. Although 90% of the subjects self-reported that genetic relationship was not relevant, their brain activity was quite different between the two groups. Bacha-Trams et al. [75] concluded that mere knowledge of a genetic relationship between interacting persons robustly modulates social cognition of the perceiver. This result is in harmony with an earlier result on sib altruism, if the different unconscious brain activities can be considered as innate reactions.

## Conclusions

We demonstrated that the self-sacrificing behaviour in monogamous and exogamous families can confer a selective advantage under the right conditions. Furthermore, some kind of evolutionary moral sense depending on the relationship has been detected in social cognition. This behaviour plays an important role nowadays in living-donor transplantation [69]. All these together call the attention that, similarly to human psychology, human morality should also be rooted in human evolution.

## Methods

We combine the methods of two research lines. The first one is theoretical population genetics. Our introduced new population genetics model is based on the mating table approach and is a generalization of the models of Hull [44] and Haldane and Jayakar [45], where the juveniles’ survival rate depends on the genotypes of their parents. The second research line is evolutionary game theory, since all individuals’ survival rate is determined by their altruistic family members. We adapt the verbal definition of evolutionary stability to genotypes, in the framework of our population genetics model.

## Additional file

Additional file 1: Supplementary information; Mathematical details and supplementary discussion. (PDF 424 kb)

## Data Availability

We have no data.

## Abbreviations

ESG: Evolutionarily stable genotype
ESS: Evolutionarily stable strategy
SI: Supplementary Information

## References

[1] Wilson EO (1971). The insect societies.

[2] Allport S (2003). A natural history of parenting: a naturalist looks at parenting in the animal world and ours.

[3] Garay J, Varga Z, Gámez M, Cabello T (2016). Sib cannibalism can be adaptive for kin. Ecol Model.

[4] Maynard Smith J (1982). Evolution and the theory of games.

[5] Maynard Smith J, Price GR (1973). The logic of animal conflict. Nature.

[6] Nowak MA (2006). Five rules for the evolution of cooperation. Science.

[7] Rand DG, Nowak MA (2013). Human cooperation. Trends Cogn Sci.

[8] Garay J, Varga Z (2011). Survivor's dilemma: defend the group or flee?. Theor. Pop. Biol..

[9] Szabó G, Fath G (2007). Evolutionary games on graphs. Phys Rep.

[10] Könnyű B, Czárán T (2013). Spatial aspects of prebiotic replicator coexistence and community stability in a surface-bound RNA world model. BMC Evol Biol.

[11] Könnyű B, Czárán T (2014). Phenotype/genotype sequence complementarity and prebiotic replicator coexistence in the metabolically coupled replicator system. BMC Evol Biol.

[12] Archetti M, Scheuring I (2012). Review: game theory of public goods in one-shot social dilemmas without assortment. J Theor Biol.

[13] Hauert C, Michor F, Nowak MA, Doebeli M (2006). Synergy and discounting of cooperation in social dilemmas. J Theor Biol.

[14] Maynard Smith J (1981). Will a sexual population evolve to an Ess?. Am Nat.

[15] Garay J, Varga Z (2003). Coincidence of ESAD and ESS in dominant-recessive hereditary systems. J Theor Biol.

[16] Hamilton WD (1964). The genetical evolution of social behaviour. I J Theor Biol.

[17] Rowthorn R (2006). The evolution of altruism between siblings: Hamilton's rule revisited. J Theor Biol.

[18] van Veelen M (2007). Hamilton’s missing link. J Theor Biol.

[19] Trivers R (1974). Parent–offspring conflict. Integr Comp Biol.

[20] Hinde CA, Johnstone RA, Kilner RM (2010). Parent-offspring conflict and coadaptation. Science.

[21] Bossan B, Hammerstein P, Koehncke A (2013). We were all young once: an intragenomic perspective on parent–offspring conflict. Proc Royal Soc B: Biol Sci.

[22] Lehmann L, Rousset F (2010). How life history and demography promote or inhibit the evolution of helping behaviours. Phil. Trans. R. Soc. B..

[23] Lion S, van Baalen M (2007). From infanticide to parental care: why spatial structure can help adults be good parents. Am Nat.

[24] Price GR (1970). Selection and covariance. Nature..

[25] Gardner A, West SA, Wild G (2011). The genetical theory of kin selection. J Evol Biol.

[26] McGlothlin Joel W., Wolf Jason B., Brodie Edmund D., Moore Allen J. (2014). Quantitative genetic versions of Hamilton's rule with empirical applications. Philosophical Transactions of the Royal Society B: Biological Sciences.

[27] Garay J, Csiszár V, Móri TF, Szilágyi A, Varga Z, Számadó S (2018). Juvenile honest food solicitation and parental investment as a life history strategy: a kin demographic selection model. PLoS One.

[28] Garay J, Számadó S, Varga Z, Szathmáry E (2018). Caring for parents: an evolutionary rationale. BMC Biol.

[29] Feldman MW, Cavalli-Sforza LL (1981). Further remarks on Darwinian selection and “altruism”. Theor. Pop. Biol..

[30] Maynard Smith J (1980). Models of the evolution of altruism. Theor. Pop. Biol..

[31] Spencer HG, Feldman MW (2005). Adaptive dynamics, game theory and evolutionary population genetics. J. Evol. Biol.

[32] Cavalli-Sforza LL, Feldman MW (1978). Darwinian selection and “altruism”. Theor. Pop. Biol.

[33] Uyenoyama M, Feldman MW (1980). Theories of kin and group selection: a population genetics perspective. Theor Pop Biol.

[34] Uyenoyama MK, Feldman MW, Mueller LD (1981). Population genetic theory of kin selection: Multiple alleles at one locus. PNAS.

[35] van Veelen M, Allen B, Hoffman M, Simon B, Veller C (2017). Hamilton's rule. J Theor Biol.

[36] Hamilton WD (1972). Altruism and related phenomena, mainly in social insects. Ann. Rev. Ecol. System..

[37] Michod RE (1980). Evolution of interactions in family-structured populations: mixed mating models. Genetics.

[38] Michod RE (1982). The theory of kin selection. Ann Rev Ecol System.

[39] Roze D, Rousset F (2004). The robustness of Hamilton’s rule with inbreeding and dominance: kin selection and fixation probabilities under partial sib mating. Am Nat.

[40] Bittles AH. Consanguinity in context. Cambridge University Press. 2012.

[41] Shepher J (1971). Mate selection among second generation kibbutz adolescents and adults: incest avoidance and negative imprinting. Arch Sex Behav.

[42] Peck JR, Feldman MW (1988). Kin selection and the evolution of monogamy. Science.

[43] Creanza N, Kolodny O, Feldman MW (2017). Cultural evolutionary theory: How culture evolves and why it matters. PNAS.

[44] Hull P (1964). Partial incompatibility not affecting total litter size in the mouse. Genetics..

[45] Haldane JBS, Jayakar SD (1965). Selection for a single pair of allelomorphs with complete replacement. J Genetics.

[46] Henrich J, Boyd R, Richerson PJ (2012). The puzzle of monogamous marriage Phil. Trans R Soc B.

[47] Dugatkin LA (2007). Inclusive fitness Theory from Darwin to Hamilton. Genetics.

[48] UNICEF Maternal mortality, February 2017, https://data.unicef.org/topic/maternal-health/maternal-mortality/ 2016.

[49] Garay, M. E. Gratitude journal: pay attention to the positive in everyday life. Correlations between trait and state levels of gratitude*.* MsC Thesis (in Hungarian). 2010.

[50] Klein, M. Envy and Gratitude: A Study of Unconscious Forces. New York: Basic Books (Reprinted 2003), Abingdon: Routledge 1957.

[51] McAdams Dan P., Bauer Jack J. (2004). Gratitude in Modern Life. The Psychology of Gratitude.

[52] Nowak MA, Roch S (2007). Upstream reciprocity and the evolution of gratitude. Proc Roy Soc B.

[53] Smith A, Pedersen EJ, Forster DE, McCullough ME, Lieberman Cooperation D (2017). The roles of interpersonal value and gratitude. Evol Hum Behav.

[54] Garay J, Garay MB (1998). Genetical reachability: when does a sexual population realize all phenotypic states?. J Math Biol.

[55] Hammerstein P (1996). Darwinian adaptation, population genetics and the streetcar theory of evolution. J Math Biol.

[56] Hofbauer J, Sigmund K (1998). Evolutionary games and population dynamics.

[57] van Veelen M (2009). Group selection, kin selection, altruism and cooperation: when inclusive fitness is right and when it can be wrong. J Theor Biol.

[58] Abbot P (2011). Inclusive fitness theory and eusociality. Nature.

[59] Nowak MA, Tarnita CE, Wilson EO (2010). The evolution of eusociality. Nature.

[60] de Vladar HP, Szathmáry E (2017). Beyond Hamilton's rule. Science.

[61] Birch J, Okasha S (2015). Kin selection and its critics. BioScience.

[62] Simon B (2014). Continuous-time models of group selection, and the dynamical insufficiency of kin selection models. J Theor Biol.

[63] Garay J, Csiszár V, Móri TF (2014). Under multilevel selection: "when shall you be neither spiteful nor envious?". J Theor Biol.

[64] Okasha S (2010). Altruism researchers must cooperate. Nature.

[65] Garay J (2009). Cooperation in defence against a predator. J Theor Biol.

[66] Schulze WS, Lubatkin MH, Dino RN (2003). Toward a theory of agency and altruism in family firms. J Business Venturing.

[67] Guttman N, Siegal G, Appel N, Bar-O G (2016). Should altruism, solidarity, or reciprocity be used as prosocial appeals? Contrasting conceptions of members of the general public and medical professionals regarding promoting organ donation. J Communication.

[68] Crowley-Matoka M (2016). Domesticating organ transplant: familial sacrifice and National Aspiration in Mexico.

[69] Davis CL, Delmonico FL (2005). Living-donor kidney transplantation: a review of the current practices for the live donor. J Am Soc Nephrol.

[70] Tanaka K, Ogura Y, Kiuchi T, Inomata Y, Uemoto S, Furukawa H (2004). Living donor liver transplantation: eastern experiences. HPB.

[71] van Overwalle F (2009). Social cognition and the brain: a meta-analysis. Hum Brain Mapp.

[72] Greene JD, Nystrom LE, Engell AD, Darley JM, Cohen JD (2004). The neural bases of cognitive conflict and control in moral judgment. Neuron.

[73] Christensen JF (2012). A Gomila. Moral dilemmas in cognitive neuroscience of moral decision-making: A principled review Neurosci Biobehav Rev.

[74] Majdandžić J, Bauer H, Windischberger C, Moser E, Engl E, Lamm C (2012). The human factor: behavioral and neural correlates of humanized perception in moral decision making. PLoS One.

[75] Bacha-Trams M, Glerean E, Dunbar R, Lahnakoski JM, Ryyppö E, Sams M, Jääskeläinen IP (2017). Differential inter-subject correlation of brain activity when kinship is a variable in moral dilemma. Sci Rep.

