# Supplementary material for: To save or not to save your family member’s life? Evolutionary stability of self-sacrificing life history strategy in monogamous sexual populations
Source: BMC Evol Biol. 2019 Jul 19;19:147. doi: 10.1186/s12862-019-1478-0 (PMC6642471; doi:10.1186/s12862-019-1478-0)
Supplement: Supplementary file 1 — Supplementary information; Mathematical details and supplementary discussion. (PDF 424 kb) [file 12862_2019_1478_MOESM1_ESM.pdf]

SUPPLEMENTARY INFORMATION

to the paper

To save or not to save your family member's life? Evolutionary stability of self-sacrificing life history strategy in monogamous sexual populations

SUPPLEMENTARY INFORMATION A

A method for the biological modelling of a moral norm

Frist we have to mention that, from the evolutionary perspective, our present knowledge on the human cooperation is rooted in the prisoner's dilemma game, which is a pure evolutionary game theoretical model (e.g. Axelrod and Hamilton 1981, Nowak and Coakley 2013, Nowak and Highfield 2011). Our motivation is related to the following general question: Is there an evolutionary root of human morality (e.g. Boehm, 2012, Darwin 1871, Garay and Móri 2011, Garay et al. 2014, 2017, 2018b, Ridley 1996, Wilson 2010)? From the perspective of theoretical biology, "evolutionary root" can be given by a simple evolutionary model, connected to a moral rule[1] (similarly to the human cooperation and prisoner's dilemma game). We note that the investigations of the evolutionary roots of human moral behaviour usually start with a social dilemma and end up with a norm that can provide some insight into the origin of morality (e.g. Nowak & Sigmund 2005, Ohtsuki & Iwasa 2006, Wilson, & Kniffin 2003). We take the opposite direction by investigating whether a moral rule can spread through Darwinian competition. To construct the corresponding models, we use the following three-step model building method (cf. Garay et al. 2018b).

[1] Evolutionary game theory is already considered to be a method to investigate the emergence of moral, see e.g. Verbeek & Morris (2018).

Step 1: Start out from a given moral rule, with a possible connection to biology. In the present paper we focus on a partial version of the golden rule[2]: "*Risk your life to save your family members, if you want them to save your life.*" This moral rule can be considered as selfless, self-sacrificing[3] and affectionate interactions, from the point of view of non-evolutionary biologists.[4]

Step 2: Give a biological formulation of the considered moral rule, i.e. introduce a purely evolutionary biological model, which is a translation of the moral rule into biological terms. Here, our biological formulation is the following: We consider a monogamous family in which the male and female differ only in sex, i.e. they are the same from all other points of view. "Self-sacrificing" in biological term means that the actors risk their own lives for the lives of their family members, i.e. the interactions change the survival rate of the family members. Furthermore, selflessness in the family can be modelled by Hamiltonian altruism between the family members.[5]

Step 3: Using the mathematical tools of evolutionary theory, we get conditions under which the biological version of a moral norm (or rule) will be evolutionarily stable. In spite of the fact that the moral commandments are unconditional rules, while in Darwinian evolution there must be a selective condition determining whether a behaviour is adaptive or not, the conditions shed a light on whether the considered moral norm has evolutionarily rationale or has not.

---

[2] The golden rule "*Do to others what you want them to do to you*." is a maxim of altruism, and it is found in many cultures and religions as a basis of human moral (e.g. Skyrms 1996).
[3] The nomenclature of "self-sacrificing" goes back to the Haldane quip: „*Legend has it that in a pub one evening Haldane told his friends that he would jump into a river and risk his life to save two brothers, but not one, and that he would jump in to save eight cousins, but not seven*."
[4] We note that our biological model complies with all major religions and non-religious humanistic traditions.
[5] The connection between altruism and human morality is already supposed (cf. Brook 2013, Dixon 2013).

The interpretation of evolutionary stability of a behaviour phenotype corresponding to a moral norm, is twofold. The given moral norm is a result of biological evolution[6], or given a moral norm, its stable maintenance against deviations is guaranteed by evolutionary stability.

SUPPLEMENTARY INFORMATION B

Evolutionary stability for interactions within a monogamous family

In a population of size N there are m types, with (relative) frequencies $\boldsymbol{y} = (y_1, y_2, \dots, y_m)$. Suppose $N/2$ couples are formed at random (N is thought large enough to allow parity problems to be disregarded). Then the probability of a prescribed pair to be coupled is $1/(N-1)$. Let $Z_{(ij)}$ denote the random number of (i,j) type families (types (i,j) and (j,i) are identical). The random sizes of surviving offspring of different families are independent and, for families of the same type, they are also identically distributed. For every family type (i,j) let $\left(\nu_{k(ij)},\ k = 1,2,\dots,m\right)$ be a vector of dependent nonnegative integer valued random variables with means $n_{k(ij)}$, resp. This is the number of surviving offspring of type $k = 1,2,\dots,m$ in a generic (i,j) type family. Finally, let $q_{i(ij)}$ denote survival probability of a type i parent in a type (i,j) family.

In the next generation the number Y_k of individuals of type k is clearly comprises the type k surviving children in families of all types, plus the surviving parents of type k. Hence,

$$E(Y_k|N,\boldsymbol{y}) = \sum_{(i,j)} n_{k(ij)} E\left(Z_{(ij)}\middle|N,\boldsymbol{y}\right) + 2q_{k(kk)} E\left(Z_{(kk)}\middle|N,\boldsymbol{y}\right) + \sum_{i \neq k} q_{k(ik)} E\left(Z_{(ik)}\middle|N,\boldsymbol{y}\right).$$

[6] The philosopher Ruse (1986, p.253) claimed that morality is "*an invention of the genes rather than humans*"; and he concludes that "*morality is a collective illusion foisted on us by our genes*." Thus, the sexual model is also interesting from philosophical point of view as well, since the asexual model can be considered as a phenotypic one.

If N is large (that is, letting N tend to infinity), then $E\left(Z_{(ij)}\middle|N,\boldsymbol{y}\right)$ is asymptotically equal to Ny_iy_j, if $i \neq j$, and to $Ny_j^2/2$, if $i = j$. Thus,

$$E(Y_k|N,\boldsymbol{y}) \sim \frac{N}{2}\left[\sum_{i=1}^{m}\sum_{j=1}^{m} n_{k(ij)}y_iy_j + 2\sum_{i=1}^{m} q_{k(ik)}y_iy_k\right].$$

(Here $a \sim b$ means asymptotic equality, that is, $\frac{a}{b} \to 1$.) At the same time the population size changes to N', with

$$E(N'|N,\boldsymbol{y}) = \sum_{k=1}^{m} E(Y_k|N,\boldsymbol{y}) \sim \frac{N}{2}\sum_{k=1}^{m}\left[\sum_{i=1}^{m}\sum_{j=1}^{m} n_{k(ij)}y_iy_j + 2\sum_{i=1}^{m} q_{k(ik)}y_iy_k\right].$$

For the sake of simplicity, in the sequel we will write = (equality sign) instead of $\sim$ (asymptotic equality).

Resident type k is ESG if $E(Y_k|N,\boldsymbol{y}) > y_kE(N'|N,\boldsymbol{y})$, provided that y_k is sufficiently close to 1; that is, the (relative) frequency of type k increases if the mutant are sufficiently rare. This is equivalent to requiring that the growth rate of the resident exceeds the average growth rate of the whole population.

Let us investigate what conditions should be imposed on the numbers $n_{k(ij)}$ and $q_{k(ik)}$ so that type 1 satisfy the definition of ESG. Suppose $y_1 = 1 - \varepsilon$, then $y_j \leq \varepsilon, 1 < j \leq m$. We have

$$\frac{2}{N}E(Y_1 - N'y_1|N,\boldsymbol{y})$$

$$= (1-y_1)\left[\sum_{i=1}^{m}\sum_{j=1}^{m} n_{1(ij)}y_iy_j + 2\sum_{i=1}^{m} q_{1(i1)}y_iy_1\right]$$

$$- y_1\sum_{k=2}^{m}\left[\sum_{i=1}^{m}\sum_{j=1}^{m} n_{k(ij)}y_iy_j + 2\sum_{i=1}^{m} q_{k(ik)}y_iy_k\right],$$

and this has to be positive if $\varepsilon > 0$ is small enough. The sums on the right-hand side have to be split according that the indices i and j are equal to, or greater than 1, because of the different order of magnitude of y_1 and y_j, $1 < j \leq m$. Here

$$(1-y_1)\left[\sum_{i=1}^{m}\sum_{j=1}^{m} n_{1(ij)}y_iy_j + 2\sum_{i=1}^{m} q_{1(i1)}y_iy_1\right]$$

$$= \left(n_{1(11)} + 2q_{1(11)}\right)\varepsilon(1-\varepsilon)^2 + 2\varepsilon\sum_{i=2}^{m}\left(n_{1(i1)} + q_{1(i1)}\right)y_i + O(\varepsilon^3),$$

and similarly,

$$y_1\sum_{k=2}^{m}\left[\sum_{i=1}^{m}\sum_{j=1}^{m} n_{k(ij)}y_iy_j + 2\sum_{i=1}^{m} q_{k(ik)}y_iy_k\right]$$

$$= (1-\varepsilon)^3\sum_{k=2}^{m} n_{k(11)} + 2(1-\varepsilon)^2\sum_{k=2}^{m}\sum_{i=2}^{m} n_{k(1i)}y_i + \sum_{k=2}^{m}\sum_{i=2}^{m}\sum_{j=2}^{m} n_{k(ij)}y_iy_j$$

$$+ 2(1-\varepsilon)^2\sum_{k=2}^{m} q_{k(1k)}y_k + 2\sum_{k=2}^{m}\sum_{i=2}^{m} q_{k(ik)}y_iy_k + O(\varepsilon^3).$$

Hence the desired inequality $\frac{2}{N}E(Y_1 - N'y_1|N,\boldsymbol{y}) > 0$ can be rewritten as

$$-(1-\varepsilon)^3\sum_{k=2}^{m} n_{k(11)} + (1-\varepsilon)^2\left(\varepsilon\big(n_{1(11)} + 2q_{1(11)}\big) - 2\sum_{k=2}^{m}\left[\sum_{i=2}^{m} n_{i(1k)} + q_{k(1k)}\right] y_k\right)$$

$$-\sum_{k=2}^{m}\sum_{i=2}^{m}\sum_{j=2}^{m} n_{k(ij)} y_i y_j - 2\sum_{k=2}^{m}\sum_{i=2}^{m} q_{k(ik)} y_i y_k + O(\varepsilon^3) > 0.$$

For the sake of convenience introduce

$$a_k = n_{1(11)} + 2q_{1(11)} - 2\left[\sum_{i=2}^{m} n_{i(1k)} + q_{k(1k)}\right], \qquad k = 1, 2, \dots, m,$$

$$c_{ij} = n_{1(i1)} + n_{1(j1)} + q_{1(i1)} + q_{1(j1)} - q_{i(ij)} - q_{j(ij)} - \sum_{k=2}^{m} n_{k(ij)}, \quad i, j = 2, \dots, m. \tag{A.1}$$

Then we have

$$\frac{2}{N} E(Y_1 - N' y_1 | N, \boldsymbol{y}) = -(1-\varepsilon)^3 \sum_{k=2}^{m} n_{k(11)} + (1-\varepsilon)^2 \sum_{k=2}^{m} a_k y_k + \sum_{i=2}^{m}\sum_{j=2}^{m} c_{ij} y_i y_j > 0. \tag{A.2}$$

Here the first term on the right-hand side is of constant order, the second one is $O(\varepsilon)$, and the last one is of order $O(\varepsilon^2)$. From this we firstly get that $\sum_{k=2}^{m} n_{k(11)} = 0$, that is, $n_{k(11)} = 0$ for all $k = 2,3, \dots, m$. From the first order term (i.e., proportional to ε) we have $\sum_{k=2}^{m} a_k y_k \geq 0$. This sum is bounded by $\varepsilon \min_{1<k} a_k$ from below, and this estimate is sharp. Therefore, a *necessary condition* for type 1 to be an ESG is

$$n_{k(11)} = 0 \text{ and } a_k \geq 0 \text{ for every } k = 2, 3, \dots, m.$$

Thus, in a family of pure type $(1,1)$, there cannot be children of any other type. In addition, the mean number of type 1 survivors (parents plus children) in families of type $(1,1)$ cannot be less

than the mean number of non-1 type survivors in families of mixed type $(1, k)$. The reason for the multiplier 2 on the right-hand side is that, though not distinguished, there are families of both types $(1, k)$ and $(k, 1)$.

If $a_k > 0$ for every $k = 2, 3, \ldots, m$, it is already sufficient for type 1 to be an ESG. In the opposite case it can happen that the right-hand side of (A.2) is at most of second order. This is the case where $y_k = O(\varepsilon^2)$ whenever $a_k > 0$. Then one has to consider the second order terms. Let $K = \{1 < k \le m :\ a_k = 0\}$. Suppose K is not empty, and $y_k = o(\varepsilon)$ if $k \notin K \cup \{1\}$, then $\sum_{k \in K} y_k = \varepsilon - o(\varepsilon)$,

$$\sum_{k=2}^{m} a_k y_k = \sum_{k \notin K \cup \{1\}} a_k y_k = o(\varepsilon),$$

and the first order term vanishes. Clearly, the above sum is always nonnegative and it can made 0 by setting $y_k = 0,\ k \notin K \cup \{1\}$, which may affect the last sum up to a term of $o(\varepsilon^2)$ at most. Thus, from the second order terms we get the following condition:

$$\sum_{i \in K} \sum_{j \in K} c_{ij} y_i y_j > 0. \qquad \text{(A.3)}$$

For the exact sufficient condition one has to compute the minimum of a quadratic form over the simplex $\Delta = \{(y_i, i \in K) \colon \forall y_i \ge 0,\ \sum_{i \in K} y_i = \varepsilon\}$, so the new condition becomes more complicated and less explicit. A more restrictive but simpler sufficient condition can easily be obtained: $c_{ij} > 0$ for every pair $i, j \in K$. Summarizing our results, we arrive at the following *sufficient condition.*

$$n_{k(11)} = 0, \text{ and } n_{1(11)} + 2q_{1(11)} \ge 2\left[\sum_{i=2}^{m} n_{i(1k)} + q_{k(1k)}\right], \quad \text{for every } k = 2, 3, \ldots, m, \text{ and} \qquad \text{(A.4)}$$

$$n_{1(i1)} + n_{1(j1)} + q_{1(i1)} + q_{1(j1)} > \sum_{k=2}^{m} n_{k(ij)} + q_{i(ij)} + q_{j(ij)} \text{ for every } i,j \in K.$$

The condition in the second line requires that the mean number of type 1 survivors (parents plus offspring) in $(i, 1)$ and $(j, 1)$ type mixed families is greater than the mean number of non-1-type survivors in (i, j) families.

In these computations we supposed that the frequencies of different mutant types can be set independently, only keeping their sum fixed. However, in certain applications there are two different kinds of mutants: *primary mutants* can appear spontaneously at any time, while *secondary mutants* can only be born to mutant–mutant couples. Hence, the densities of primary mutant types can be set arbitrarily (not exceeding ε in sum), but secondary mutants must have smaller, $O(\varepsilon^2)$ densities. Let us investigate how our sufficient conditions change in this more general case.

Let P and S denote the sets of primary and secondary mutant types, resp., thus $\{2,3,\dots,m\} = P \cup S$. Then we still have to require $n_{k(11)} = 0$ for all mutant types $k = 2,3,\dots,m$, but the first order term in the expansion of $\frac{2}{N}E(Y_1 - N'y_1|N, \boldsymbol{y})$ reduces to $\sum_{k\in P} a_k y_k$. Hence, the first order conditions are

$a_k \geq 0$ for every $k \in P$ (necessary),

$a_k > 0$ for every $k \in P$ (sufficient).

Regarding the computations, secondary mutants are similar to mutant types $k \notin K$ in the simpler model. Therefore, the definition of K is somewhat modified: $K = \{k \in P : a_k = 0\}$. If $K \neq \emptyset$ then the second order condition (A.3) remains valid, but in the second order term an additional sum $\sum_{k\in S} a_k y_k$ appears at this point (because this sum has now become of second order). We may require that $a_k \geq 0$ for all $k \in S$, but sometimes it proves to be too crude. In

order to derive sharper results, we first need to estimate how large the proportion of secondary mutants can be.

In the beginning, there are no secondary mutants at all. Suppose the proportion of secondary mutants is less than $C\varepsilon^2$ at a given moment, where ε is the proportion of all mutants. Secondary mutants of the next generation can be the children of mutant-mutant couples, or surviving parents. In the former case we can only consider primary-primary families, and in the latter, resident-secondary mutant ones, because all other possibilities lead to third order terms. Hence the asymptotic (as $N \to \infty$) proportion of secondary mutants in the next generation is

$$\frac{1}{E(N'|N,\boldsymbol{y})}\sum_{k\in S} E(Y_k|N,\boldsymbol{y}) = \frac{N}{2E(N'|N,\boldsymbol{y})}\sum_{k\in S}\left[\sum_{i\in P}\sum_{j\in P} n_{k(ij)}y_i y_j + 2q_{k(1k)}y_k\right] + O(\varepsilon^3)$$

$$\leq \frac{N}{2E(N'|N,\boldsymbol{y})}\left[\sum_{i\in P}\sum_{j\in P}\sum_{k\in S} n_{k(ij)}\, y_i y_j + 2C\max_{k\in S} q_{k(1k)}\,\varepsilon^2\right] + O(\varepsilon^3)$$

$$= \frac{N}{2E(N'|N,\boldsymbol{y})}\sum_{i\in P}\sum_{j\in P}\left[\sum_{k\in S} n_{k(ij)} + 2C\max_{k\in S} q_{k(1k)}\right] y_i y_j + O(\varepsilon^3).$$

Similarly, the proportion of all mutants is

$$\frac{1}{E(N'|N,\boldsymbol{y})}\sum_{k=2}^{m} E(Y_k|N,\boldsymbol{y}) = \frac{N}{2E(N'|N,\boldsymbol{y})}\sum_{k=2}^{m}\left[\sum_{i=1}^{m}\sum_{j=1}^{m} n_{k(ij)}y_i y_j + 2\sum_{i=1}^{m} q_{k(ik)}y_i y_k\right].$$

Remembering our supposition $n_{k(11)} = 0$ for all $k = 2,3,\dots,m$, we can see that for the principal term in the double sum it is sufficient to consider the summands with $i \in P, j = 1$ or vice versa, and the last sum can also be replaced with its first term, and $k \in P$ can also be supposed there. Thus,

$$\frac{1}{E(N'|N,\boldsymbol{y})}\sum_{k=2}^{m} E(Y_k|N,\boldsymbol{y}) = \frac{N}{E(N'|N,\boldsymbol{y})}\sum_{i\in P}\left[\sum_{k=2}^{m} n_{k(i1)} + q_{i(i1)}\right] y_i + O(\varepsilon^2).$$

Introduce

$$f = 2\max_{k\in S} q_{k(1k)}\,, \qquad g_{ij} = \sum_{k\in S} n_{k(ij)},$$

$$h_i = 2\left[\sum_{k=2}^{m} n_{k(i1)} + q_{i(i1)}\right] = n_{1(11)} + 2q_{1(11)} - a_i, \tag{A.5}$$

then the proportions of secondary resp. all mutants are

$$\le \frac{N}{2E(N'|N,\boldsymbol{y})}\sum_{i\in P}\sum_{j\in P}\big(g_{ij} + fC\big)y_i y_j + O(\varepsilon^3), \text{ and } = \frac{N}{2E(N'|N,\boldsymbol{y})}\sum_{i\in P} h_i y_i + O(\varepsilon^2),$$

respectively. Here $E(N'|N,\boldsymbol{y})$ is asymptotically equal to

$$E(Y_1|N,\boldsymbol{y}) = \frac{N}{2}\big(n_{1(11)} + 2q_{1(11)} + o(1)\big),$$

hence upper estimate $C\varepsilon^2$ can be proved by induction if

$$\big(n_{1(11)} + 2q_{1(11)}\big)\sum_{i\in P}\sum_{j\in P}\big(g_{ij} + fC\big)y_i y_j < C\left[\sum_{i\in P} h_i y_i\right]^2 = C\sum_{i\in P}\sum_{j\in P} h_i h_j y_i y_j$$

for arbitrary nonnegative numbers $y_i, i \in P$. This is satisfied if for every $i,j \in P$

$$\big(n_{1(11)} + 2q_{1(11)}\big)\big(g_{ij} + fC\big) < Ch_i h_j,$$

that is,

$$\big(n_{1(11)} + 2q_{1(11)}\big)g_{ij} < \big[h_i h_j - \big(n_{1(11)} + 2q_{1(11)}\big)f\big]C.$$

It follows that for every $C > C'$ where

$$C' = \big(n_{1(11)} + 2q_{1(11)}\big)\max\Big\{\big[h_i h_j - \big(n_{1(11)} + 2q_{1(11)}\big)f\big]^{-1} g_{ij} : \ i,j \in P\Big\}, \tag{A.6}$$

the proportion of secondary mutants is less than $C\varepsilon^2$, provided ε, the proportion of all mutants, is sufficiently small. (Of course, C' should be positive, otherwise no upper bound of the form $C\varepsilon^2$ can be valid.) Therefore,

$$\sum_{k\in S} a_k y_k \ge C\min\{0, a_k, k \in S\}\,\varepsilon^2,$$

and consequently, the second order term is positive if, instead of the last line of (A.4), inequality

$$C'\min\{0, a_k, k \in S\} + n_{1(i1)} + q_{1(i1)} + n_{1(j1)} + q_{1(j1)} - \sum_{k=2}^{m} n_{k(ij)} + q_{i(ij)} + q_{j(ij)} > 0$$

holds for every $i, j \in K$.

Finally, we conclude with the following theorem.

Theorem 1

Let $P \subset \{2,3,\dots,m\}$ *and* $S = \{2,3,\dots,m\} \setminus P$ *denote the set of primary and secondary mutant types, resp. Type* 1 *is evolutionarily stable if the following conditions hold.*

(i) $n_{k(11)} = 0$ *for every* $k = 2, 3, \dots, m$.

(ii) $a_k \geq 0$ *for every* $k \in P$, *where* a_k *is defined in* (A.1).

(iii) *Let* $K = \{k \in P : a_k = 0\}$. *If* $K \neq \emptyset$ *then*

$$C'\min\{0, a_k, k \in S\} + n_{1(i1)} + n_{1(j1)} + q_{1(i1)} + q_{1(j1)}$$

$$> \sum_{k=2}^{m} n_{k(ij)} + q_{i(ij)} + q_{j(ij)}$$

for every $i, j \in K$, *where* C' *is defined in* (A.5) and (A.6).

Remark. In the particular case where $K = P$, formula (A.6) becomes much simpler, namely,

$$C' = \frac{\max_{i,j \in P} g_{ij}}{n_{1(11)} + 2q_{1(11)} - 2\max_{k \in S} q_{k(1k)}}.$$

Application. Consider the case where there are 3 types: $[a, a]$ (resident), $[a, A]$ (primary mutant), and $[A, A]$ (secondary mutant). Genotype distribution among the offspring follows the Mendelian rules. We suppose that the average offspring size is equal in all family types, say n. Then $P = \{2\}$ and $S = \{3\}$, moreover,

$$n_{2(11)} = n_{3(11)} = n_{3(12)} = n_{1(13)} = n_{3(13)} = n_{1(23)} = n_{1(33)} = n_{2(33)} = 0,$$

$$a_2 = n_{1(11)} + 2q_{1(11)} - 2\big(n_{2(12)} + q_{2(12)}\big),$$

$$a_3 = n_{1(11)} + 2q_{1(11)} - 2\big(n_{2(13)} + q_{3(13)}\big),$$

and condition **(iii)** reads as follows. If $a_2 = 0$, then

$$\begin{aligned} &\text{either } \ a_3 \geq 0, \ \text{ and } \ n_{1(11)} + 2q_{1(11)} - n_{2(22)} - n_{3(22)} - 2q_{2(22)} > 0, \\ &\text{or } \ a_3 < 0, \ \text{ and } \ C'a_3 + \ 2n_{1(12)} + 2q_{1(12)} - n_{2(22)} - n_{3(22)} - 2q_{2(22)} > 0, \end{aligned} \tag{A.7}$$

where

$$C' = \frac{n_{3(22)}}{n_{1(11)} + 2q_{1(11)} - 2q_{3(13)}}.$$

This is the sufficient condition for the resident $[a, a]$ to be an ESG. Note that here the same inequality with $\geq$ is already necessary, because P and Q are singletons, so the minimum of the left-hand side of (A.3) over the simplex Δ is attained, and all estimations in the derivation of C' are valid with equality.

B.1 Suppose first that **the mutation is recessive**. Then $n_{1(12)} = n_{2(12)} = n_{1(11)}/2$, and $q_{1(11)} = q_{1(12)} = q_{2(12)}$. Hence $a_2 = 0$, and $K = \{2\}$. We have to check the second order conditions **(iii).**

Let us apply this to our additive models. Note that parent-offspring and sibling-sibling interactions are supposed to take place before natural selection, thus all offspring of suitable type are involved, not only those who survive.

B.1.1 Firstly, let a stand for **grateful**, A for non-grateful behaviour. A grateful sibling increases the survival probability of one of its parents by γ, while its own survival probability decreases by c_g. Hence we have $n_{2(22)} = \frac{1}{2} n_{1(11)}$, $n_{3(22)} = \frac{1}{4}\big(n_{1(11)} + nc_g\big)$, $q_{2(22)} = q_{1(11)} - \frac{1}{8}\, n\gamma$, $q_{3(13)} = q_{1(11)}$. Thus, $C'a_3 = -n_{3(22)}$, and grateful behaviour is ESG if $\gamma > 2c_g$.

B.1.2 Next, let a denote **altruistic**, A non-altruistic behaviour. An altruistic sibling increases the survival probability of each of its $n-1$ siblings by b, while its own survival probability decreases by $(n-1)c_a$. Let (η_1, η_2, η_3) denote the number of type 1, type 2, and type 3 siblings

in a (2,2) family. This random vector has multinomial distribution with parameters $n, \left(\frac{1}{4}, \frac{1}{2}, \frac{1}{4}\right)$. Therefore, the survival probability of an altruistic sibling in a (2,2) type family is less by $\eta_3 b$ than that in a (1,1) type family. At the same time, the survival probability of a non-altruistic sibling increases by $(n-1)c_a$, but decreases by $(\eta_3 - 1)b$ (with respect to siblings in (1,1) type families). Hence,

$$n_{2(22)} = \frac{1}{2} n_{1(11)} - E(\eta_2 \eta_3)b = \frac{1}{2} n_{1(11)} - \frac{1}{8} n(n-1)b,$$

$$n_{3(22)} = \frac{1}{4} n_{1(11)} + E\eta_3(n-1)c_a - E\big(\eta_3(\eta_3 - 1)\big)b$$

$$= \frac{1}{4} n_{1(11)} + \frac{1}{4} n(n-1)c_a - \frac{1}{16} n(n-1)b.$$

while $q_{1(11)} = q_{2(22)} = q_{3(22)} = q_{3(13)}$ here. Thus, $C' a_3 = -n_{3(22)}$ again, and altruistic behaviour is ESG if $b > 2c_a$.

B.1.3 Finally, in the case where a denotes **provider**, A non-provider behaviour, the survival rate of a provider, resp. non-provider parent is q_1 and q_2, while the survival probabilities of the siblings in families with exactly 0, 1, or 2 provider parents are π_0, π_1, and π_2, resp. Obviously, $q_1 < q_2$, and $\pi_0 < \pi_1 < \pi_2$. Hence family types (1,1), (1,2), (2,2) are equivalent: $n_{1(11)} = n\pi_2$, $n_{1(12)} = n_{2(12)} = n_{2(22)} = \frac{1}{2} n\pi_2$, $n_{1(22)} = n_{3(22)} = \frac{1}{4} n\pi_2$. Furthermore, $n_{2(13)} = n\pi_1$, $q_{1(11)} = q_{1(12)} = q_{2(22)} = q_1$, and $q_{3(13)} = q_2$. Thus, $a_3 = -[n(2\pi_1 - \pi_2) + 2(q_2 - q_1)]$, which can be positive or negative as well. Now, we have to require that $2(q_2 - q_1) < n\pi_2$, and then

$$C' = \frac{n\pi_2}{4n\pi_2 - 8(q_2 - q_1)}.$$

If $n(\pi_2 - 2\pi_1) > 2(q_2 - q_1)$, the denominator of C' is positive, and condition **(iii)** simplifies to $\frac{1}{4} n\pi_2 > 0$, which is always true. If $n(\pi_2 - 2\pi_1) \le 2(q_2 - q_1)$, then condition **(iii)** leads to inequality

$$\frac{n\pi_2[n(\pi_2-2\pi_1)-2(q_2-q_1)]}{4n\pi_2-8(q_2-q_1)}+\frac{n\pi_2}{4}=\frac{n\pi_2[n(\pi_2-\pi_1)-2(q_2-q_1)]}{2n\pi_2-4(q_2-q_1)}>0,$$

that is, $n(\pi_2-\pi_1)>2(q_2-q_1)$. This already implies that the denominator of C' is positive. Summarizing, we obtain that the provider behaviour is ESG if $n(\pi_2-\pi_1)>2(q_2-q_1)$.

B.2 Let us turn to the case of **dominant mutation**. This time $n_{2(22)}=\ 2n_{3(22)}$.

B.2.1 Grateful resident. Here $n_{1(12)}=\ \frac{1}{2}n_{1(11)}$, $n_{2(12)}=n_{2(22)}=\frac{1}{2}n_{1(11)}+\frac{1}{2}nc_g$, $n_{2(13)}=2n_{2(12)}$; furthermore, $q_{1(12)}=q_{2(12)}=q_{1(11)}-\frac{1}{4}n\gamma$, $q_{2(22)}=q_{1(11)}-\frac{3}{8}n\gamma$, $q_{3(13)}=q_{1(11)}-\frac{1}{2}n\gamma$. From these, $a_2=n_{1(11)}+2q_{1(11)}-n_{1(11)}-nc_g-2q_{1(11)}+\frac{1}{2}n\gamma=\frac{1}{2}n(\gamma-2c_g)$. Thus, the first order sufficient condition for the grateful behaviour to be an ESG is the same as in the case of recessive mutation: $\gamma>2c_g$.

Suppose $\gamma=2c_g$, that is, $a_2=0$, and $K=\{2\}$. Let us check the second order condition **(iii)**. We have

$$a_3=n_{1(11)}+2q_{1(11)}-2n_{1(11)}-2nc_g-2q_{1(11)}+n\gamma=-n_{1(11)}<0,$$

$$C'=\frac{\frac{1}{4}\left(n_{1(11)}+nc_g\right)}{n_{1(11)}+n\gamma}=\frac{n_{1(11)}+nc_g}{4\left(n_{1(11)}+2nc_g\right)},$$

hence the second order condition is tantamount to the following inequality,

$$C'a_3+2\left(n_{1(12)}+q_{1(12)}\right)-n_{2(22)}-n_{3(22)}-2q_{2(22)}>0.$$

That is, by using that $\gamma=2c_g$,

$$-\frac{n_{1(11)}\left(n_{1(11)}+nc_g\right)}{4\left(n_{1(11)}+2nc_g\right)}+n_{1(11)}+2q_{1(11)}-nc_g-\frac{3}{4}n_{1(11)}-\frac{3}{4}nc_g-2q_{1(11)}+\frac{3}{2}nc_g>0.$$

Multiplying with the denominator, after some calculus we obtain that $-2(nc_g)^2>0$, which is never fulfilled. What is more, strict inequality holds in the opposite direction, which shows that the first order condition $\gamma>2c_g$ is *sufficient and necessary*.

B.2.2 Altruistic resident. Introduce η as the number of type 2 offspring born in a family of type $(1,2)$. It has binomial distribution with parameters n and $\frac{1}{2}$. The same holds for the number of type 2 offspring born in a $(2,2)$ family. This time

$$n_{1(12)} = \frac{1}{2}n_{1(11)} - E\big(\eta(n-\eta)\big)b = \frac{1}{2}n_{1(11)} - \frac{1}{4}n(n-1)b,$$

$$n_{2(12)} = \frac{1}{2}n_{1(11)} + \frac{1}{2}n(n-1)c_a - E\big(\eta(\eta-1)\big)b$$

$$= \frac{1}{2}n_{1(11)} + \frac{1}{2}n(n-1)c_a - \frac{1}{4}n(n-1)b,$$

$$n_{2(22)} = \frac{1}{2}n_{1(11)} + \frac{1}{2}n(n-1)c_a - E\big(\eta(n-1-\eta)\big)b$$

$$= \frac{1}{2}n_{1(11)} + \frac{1}{2}n(n-1)c_a - \frac{3}{8}n(n-1)b,$$

$$n_{2(13)} = n_{1(11)} + n(n-1)(c_a - b).$$

Since altruism among siblings does not affect parents' survival rates, all $q_{i(ij)}$ are equal. Hence,

$$a_2 = n_{1(11)} - 2n_{2(12)} = \frac{1}{2}n(n-1)(b-2c_a).$$

Consequently, the first order sufficient condition for the altruistic behaviour to be an ESG in the case of dominant mutation is the same as the second order one in the case of recessive mutation: $b > 2c_a$.

Suppose $b = 2c_a$, thus $a_2 = 0$, and $K = \{2\}$. Then $a_3 = n_{1(11)} - 2n_{2(13)} = -n_{1(11)} + 2n(n-1)c_a$, its sign can be arbitrary. Let us compute C'.

$$C' = \frac{\frac{1}{4}\big(n_{1(11)} + n(n-1)c_a - \frac{3}{4}n(n-1)b\big)}{n_{1(11)}} = \frac{n_{1(11)} - \frac{1}{2}n(n-1)c_a}{4n_{1(11)}}.$$

Now, there are two possibilities. Firstly, if $a_3 \geq 0$, that is, $n_{1(11)} \leq 2n(n-1)c_a$, then the second order condition would be

$$2n_{1(12)} - n_{2(22)} - n_{3(22)} = n_{1(11)} - n(n-1)c_a - \frac{3}{4}n_{1(11)} + \frac{3}{8}n(n-1)c_a$$

$$= \frac{1}{4}n_{1(11)} - \frac{5}{8}n(n-1)c_a > 0.$$

This contradicts the supposition.

Secondly, if $a_3 < 0$, that is, $n_{1(11)} > 2n(n-1)c_a$, then we need

$$C'a_3 + \frac{1}{4}n_{1(11)} - \frac{5}{8}n(n-1)c_a > 0,$$

and multiplying by the denominator of C' we get to the following inequality:

$$\left(n_{1(11)} - \tfrac{1}{2}n(n-1)c_a\right)\left(-n_{1(11)} + 2n(n-1)c_a\right) + n_{1(11)}\left(n_{1(11)} - \tfrac{5}{2}n(n-1)c_a\right)$$

$$= -(n(n-1)c_a)^2 > 0.$$

Since this inequality holds in the opposite direction, it shows that the first order condition $b > 2c_a$ is both *sufficient and necessary*.

B.2.3 Provider resident. Now $n_{1(11)} = n\pi_2$, $n_{1(12)} = n_{2(12)} = \frac{1}{2}n\pi_1$, $n_{2(22)} = \frac{1}{2}n\pi_0$, $n_{2(13)} = n\pi_1$, $q_{1(11)} = q_{1(12)} = q_{1(13)} = q_1$, and $q_{2(12)} = q_{2(22)} = q_{3(13)} = q_2$.

By that, $a_2 = n(\pi_2 - \pi_1) - 2(q_2 - q_1)$, hence the first order condition for the provider behaviour to be ESG is $n(\pi_2 - \pi_1) > 2(q_2 - q_1)$ again.

Suppose $n(\pi_2 - \pi_1) = 2(q_2 - q_1)$, then $a_3 = -[n(2\pi_1 - \pi_2) + 2(q_2 - q_1)] = -n\pi_1 < 0$, and

$$C' = \frac{\frac{1}{4}n\pi_0}{n\pi_2 - 2(q_2 - q_1)} = \frac{\pi_0}{4\pi_1}.$$

Thus the second order condition is $0 < n(\pi_1 - \pi_0) - 2(q_2 - q_1) = n(2\pi_1 - \pi_0 - \pi_2)$, that is,

$$\pi_1 > \frac{\pi_0 + \pi_2}{2}.$$

In other words, the benefit of two provider parents is less than the double of the benefit of a single provider.

Summarizing, the provider behaviour is ESG if either $n(\pi_2-\pi_1)>2(q_2-q_1)$, or $n(\pi_2-\pi_1)=2(q_2-q_1)$ and $\pi_1>\frac{1}{2}(\pi_0+\pi_2)$.

SUPPLEMENTARY INFORMATION C

Genotype dynamics

Let N be the population size of the whole parent population and $\frac{N}{2}$ the number of parent pairs and recall that $y=(y_1,y_2,y_3)$ is the frequency vector of genotypes G_1, G_2 and G_3 in the present generation. Based on Table 1 (mating table) of Section 3.1, the total numbers of individuals of genotypes G_1, G_2 and G_3 in the next generation, are the following:

$$Y_1(y)=\frac{N}{2}\left(y_1^2(2q_{1(11)}+n_{1(11)})+2y_1y_2(q_{1(12)}+n_{1(12)})+2y_1y_3q_{1(13)}+y_2^2n_{1(22)}\right),$$

$$Y_2(y)=\frac{N}{2}\left(2y_1y_2(q_{2(12)}+n_{2(12)})+2y_1y_3n_{2(13)}+y_2^2(2q_{2(22)}+n_{2(22)})+2y_2y_3(q_{2(23)}+n_{2(23)})\right),$$

$$Y_3(y)=\frac{N}{2}\left(2y_1y_3q_{3(13)}+y_2^2n_{3(22)}+2y_2y_3(q_{3(23)}+n_{3(23)})+y_3^2(2q_{3(33)}+n_{3(33)})\right).$$

We introduce the *genotypic reproduction rate* of genotypes G_1, G_2 and G_3 as a follows

$$w_1(y)=y_1(2q_{1(11)}+n_{1(11)})+2y_2(q_{1(12)}+n_{1(12)})+2y_3q_{1(13)},$$

$$w_2(y)=2y_1(q_{2(12)}+n_{2(12)})+y_2(2q_{2(22)}+n_{2(22)})+2y_3(q_{2(23)}+n_{2(23)}),$$

$$w_3(y)=2y_1q_{3(13)}+2y_2(q_{3(23)}+n_{3(23)})+y_3(2q_{3(33)}+n_{3(33)}),$$

and the *Mendelian production rates* of genotypes

$$M_1(y)=y_2^2n_{1(22)},$$

$$M_2(y)=2y_1y_3n_{2(13)},$$

$$M_3(y)=y_2^2n_{3(22)}.$$

Using this notation we have

$$Y_i(y) = \frac{N}{2}\left(y_i\, w_i(y) + M_i(y)\right).$$

The average growth rate of the population is

$$\overline{W}(y) = \frac{2}{N}\sum_i Y_i(y) = \sum_i \left(y_i w_i(y) + M_i(y)\right).$$

In order to make $\overline{W}$ strictly positive on the standard two-dimensional simplex

$$\Delta = \{y = (y_1, y_2, y_3) \in \mathbb{R}^3 : 0 \leq y_1, y_2, y_3 \leq 1, \text{and } y_1 + y_2 + y_3 = 1\},$$

we assume that $q_{i(jk)} > 0$ and $n_{i(jk)} > 0$ for all $i, j, k \in \{1, 2, 3\}$. This in fact implies that $w_i(y) > 0$ and $M_i(y) \geq 0$ for all $y \in \Delta$ and $i = 1, 2, 3$. In particular, $\overline{W}(y) > 0$ for all $y \in \Delta$. Now, let us follow the standard biological reasoning applied for the derivation of the replicator dynamics (see e.g. Maynard Smith 1982 Appendix D). In the next generation the genotype distribution is

$$y_i(t+1) = \frac{Y_i(y(t))}{\sum Y_j(y(t))}.$$

Since

$$\begin{aligned} y_i(t+1) - y_i(t) &= \frac{y_i w_i(y(t)) + M_i(y(t))}{\sum_i \left(y_i w_i(y(t)) + M_i(y(t))\right)} - y_i \\ &= \frac{y_i w_i(y(t)) + M_i(y(t)) - y_i \sum_i \left(y_i w_i(y(t)) + M_i(y(t))\right)}{\overline{W}(y(t))}, \end{aligned}$$

for the continuous-time model we get

$$\dot{y}_i = \frac{y_i w_i + M_i - y_i \sum_j \left(y_j w_j + M_j\right)}{\overline{W}}.$$

Clearly, $\sum_i \dot{y}_i = 0$ by $\sum_i y_i = 1$. Since multiplying the right-hand side of the above equation by $\overline{W} > 0$ does not imply an essential change[7], we get the following *genotype dynamics*:

[7] This operation does not affect the orbits, only changes the velocity (see e.g. Hofbauer and Sigmund 1998, pp. 118-119).

$$\dot{y}_i = y_i\left(w_i - \sum_{j=1}^{3} y_j w_j\right) + M_i - y_i \sum_{j=1}^{3} M_{j,}\ i = 1,2,3. \qquad \text{(B.1)}$$

Observe that the genotype dynamics is quite different from the standard replicator dynamics, since according to the Mendelian inheritance, the offspring genotypes can be different from their parents' genotypes. In contrast to the standard replication dynamics, the simplex Δ is only positively invariant under the above dynamics. Obviously, $y^* = (1,0,0)$ and $y^{***} = (0,0,1)$ are rest points of the genotype dynamics but $y_{**} = (0,1,0)$, the second corner of Δ is not a rest point. As we shall see next, the boundary of Δ (with the exception of the two corner rest points) is carried instantaneously by the dynamics into the interior of Δ.

For convenience, system (B.1) can be reformulated as

$$\dot{y}_1 = y_1(ay_1 + by_2 + cy_3) + \alpha y_2^2 - y_1 Q(y),$$

$$\dot{y}_2 = y_2(dy_1 + ey_2 + fy_3) + \beta y_1 y_3 - y_2 Q(y),$$

$$\dot{y}_3 = y_3(gy_1 + hy_2 + iy_3) + \gamma y_2^2 - y_3 Q(y),$$

where parameters $a, b, \ldots, h, i$ and α, β, γ are all positive and

$$Q(y) = y_1(ay_1 + by_2 + cy_3) + y_2(dy_1 + ey_2 + fy_3) + y_3(gy_1 + hy_2 + iy_3) + (\alpha + \gamma)y_2^2 + \beta y_1 y_3 .$$

We claim that, for each $y \in \partial\Delta\ \{y^*, y^{***}\}$, the trajectory through y intersects $\partial\Delta$ transversely, and remains in $\Delta \backslash \partial\Delta$ for all $t > 0$. (At $y = y_{**} \in \partial\Delta$, the intersection is transverse to both elongated edges of $\partial\Delta$ containing y_{**}).

In fact, by a simple Lyapunov argument, it is enough to check that the following three scalar products are positive. More precisely, our task is reduced to prove that

$$(\dot{y}_1, \dot{y}_2, \dot{y}_3) \cdot \left[(1,0,0) - \left(0, \tfrac{1}{2}, \tfrac{1}{2}\right)\right] > 0 \ \text{ whenever } \ y_1 = 0,\ y_2 + y_3 = 1, \text{and } 0 < y_2 \le 1,$$

$$(\dot{y}_1, \dot{y}_2, \dot{y}_3) \cdot \left[(0,1,0) - \left(\tfrac{1}{2}, 0, \tfrac{1}{2}\right)\right] > 0 \ \text{ whenever } \ y_2 = 0,\ y_3 + y_1 = 1, \text{and } 0 < y_1 < 1,$$

$$(\dot{y}_1, \dot{y}_2, \dot{y}_3) \cdot \left[(1,0,0) - \left(\tfrac{1}{2}, \tfrac{1}{2}, 0\right)\right] > 0 \ \text{ whenever } \ y_3 = 0,\ y_1 + y_2 = 1, \text{and } 0 < y_2 \le 1.$$

Let us concentrate on the first inequality. Then

$$Q(y) = y_2(ey_2 + fy_3) + y_3(hy_2 + iy_3) + (\alpha + \gamma)y_2^2$$

and

$$\dot{y}_1 = \alpha y_2^2,\ \ \dot{y}_2 = y_2(ey_2 + fy_3) - y_2 Q(y),\ \ \dot{y}_3 = y_3(hy_2 + iy_3) + \gamma y_2^2 - y_3 Q(y).$$

All in all, we obtain that

$$(\dot{y}_1, \dot{y}_2, \dot{y}_3) \cdot \left[(1,0,0) - \left(0, \tfrac{1}{2}, \tfrac{1}{2}\right)\right] = \alpha y_2^2 - \tfrac{1}{2}\gamma y_2^2 + \tfrac{1}{2}(\alpha + \gamma)y_2^2 = \tfrac{3}{2}\alpha y_2^2.$$

Similarly,

$$(\dot{y}_1, \dot{y}_2, \dot{y}_3) \cdot \left[(0,1,0) - \left(\tfrac{1}{2}, 0, \tfrac{1}{2}\right)\right] = \tfrac{3}{2}\beta y_1 y_3,\ \ (\dot{y}_1, \dot{y}_2, \dot{y}_3) \cdot \left[(1,0,0) - \left(\tfrac{1}{2}, \tfrac{1}{2}, 0\right)\right] = \tfrac{3}{2}\gamma y_2^2,$$

and we are done.

The Darwinian tenet claims: if the fitness (average growth rate) of a type is higher than that of the whole population, then it will spread. The basic idea of evolutionary stability claims more: If the fitness of the resident type is higher than that of the rare mutants, then the resident will be fixed. The following Theorem 2, in general terms, claims that the basic idea of evolutionary stability is generally true in the framework of the differential equation model, if there exist a neighbourhood of the homozygote equilibrium where the resident overperforms the mutant.

Theorem 2. *Consider a system of differential equations*

$$\dot{y}_1 = f_1(y_1, y_2 y_3),\ \dot{y}_2 = f_2(y_1, y_2 y_3),\ \dot{y}_3 = f_1(y_1, y_2 y_3), \tag{B.2}$$

on the standard two-dimensional simplex

$$\Delta = \{y = (y_1, y_2, y_3) \in \mathbb{R}^3 : 0 \le y_1, y_2, y_3 \le 1, \text{and } y_1 + y_2 + y_3 = 1\}.$$

Let $B(y^*, r)$ *denote the tree-dimensional open ball of radius* $r > 0$*, centred at* $y^* = (1,0,0) \in \Delta$*. We assume that* Δ *is positively invariant with respect to the dynamics governed by* (B.2). *In particular, we assume that* $\dot{y}_1 + \dot{y}_2 + \dot{y}_3 = f_1 + f_2 + f_3 = 0$ *on* Δ*. In addition, we assume that* $y^* = (1,0,0) \in \Delta$ *is an equilibrium point of the dynamics. Last but not least, we assume for some*

$0 < r_0 < 1$ *that* $f_1(y_1, y_2 y_3) > 0$, *whenever* $(y_1, y_2, y_3) \in B(y^*, r_0) \cap (\Delta \setminus \{y^*\})$. *Then the equilibrium point* $y^* = (1,0,0) \in \Delta$ *is locally asymptotically stable for the dynamics governed by* (B.2) *on* Δ.

Proof: In fact, for $r > 0$ small enough, the $0 < \tau < r$ family of closed segments of the form

$$\{(1-\tau, 0, \tau)\lambda + ((1-\tau, \tau, 0)(1-\lambda) \in \mathbb{R}^3 : 0 \leq \lambda \leq 1\} \subset \Delta$$

constitute the level surface of a strict Lyapunov function near $y^* = (1,0,0)$.

Observe that Theorem 2 implies that when the resident is recessive then the resident recessive homozygote is locally asymptotically stable respect to the genotype dynamics. Indeed, the first order condition in Appendix A implies that near the equilibrium $y^* = (1,0,0)$ inequality

$$\frac{y_1 w_1 + M_1}{y_1} > \sum_{j=1}^{3} \left(y_j w_j + M_j \right)$$

holds, which corresponds to condition $f_1(y_1, y_2 y_3) > 0$ of the above Theorem 2.

References

Axelrod, R.; Hamilton, W. 1981. The evolution of cooperation. *Science* **211(4489)**: 1390–1396. doi:10.1126/science.7466396

Boehm, C. 2012. *Moral origins: The evolution of virtue, altruism, and shame.* New York: Basic Books. ISBN 978-0465020485

Brook, J.H. 2013. Ready to Aid One Another": Darwin on Nature, God, and Cooperation In: Nowak, M.A., Coakley, S. (Eds) *Evolution, Games, and God: The Principle of Cooperation*, Cambridge, MA: Harvard Univ. Press, pp. 37-59.

Darwin, C. 1871. *The Descent of Man, and Selection in Relation to Sex.* London: John Murray. ISBN 0801420857

Dixon T. 2013. Altruism: Morals from history. In: Nowak, M.A., Coakley, S. (Eds) *Evolution, Games, and God: The Principle of Cooperation*, Cambridge, MA: Harvard Univ. Press, pp.60–81.

Garay, J., Csiszár, V., Móri, T.F. (2014). Under multilevel selection: "When shall you be neither spiteful nor envious?" *J. Theor. Biol.* **340**: 73–84. doi:10.1016/j.jtbi.2013.08.031

Garay, J., Móri, T.F. (2011). Is envy one of the possible evolutionary roots of charity?

Garay, J., Számadó, S., Varga, Z., Szathmáry E. (2018b). Caring for parents: an evolutionary rationale. BMC Biology, **16**:53 doi.org/10.1186/s12915-018-0519-2

Nowak, M.A., Coakley, S. (Eds) (2013) *Evolution, Games, and God: The Principle of Cooperation*, Cambridge, MA: Harvard Univ. Press. ISBN 978-0674047976

Nowak, M.A, Highfield, R. (2011). *SuperCooperators: Why We Need Each Other to Succeed.* New York: Simon & Schuster. ISBN 978-1451626636

Nowak, M.A., Sigmund K. (2005) Evolution of indirect reciprocity. Nature 437:1291–1298 doi.org/10.1038/nature04131.

Ohtsuki, H., Iwasa Y. (2006) The leading eight: Social norms that can maintain cooperation by indirect reciprocity. *J. Theor. Biol.* **239**, 435-444. doi.org/10.1016/j.jtbi.2005.08.008

Ridley, M. (1996). *The Origins of Virtue: Human Instincts and the Evolution of Cooperation.* London: Viking (Penguin Books). ISBN 978-0140264456

Ruse, M. (1986). *Taking Darwin Seriously: A Naturalistic Approach to Philosophy*. Oxford: Blackwell. ISBN 978-0631135425

Skyrms, B. (1996). *Evolution of the Social Contract*. Cambridge: Cambridge University Press. ISBN 978-0521555838

Verbeek, B., Morris, C. "Game Theory and Ethics", *The Stanford Encyclopedia of Philosophy* (Fall 2018 Edition), Edward N. Zalta (ed.), https://seop.illc.uva.nl/entries/game-ethics/

Wilson, D.S. (2010). *Darwin's Cathedral: Evolution, Religion, and the Nature of Society*. Chicago, IL: Univ. Chicago Press. ISBN 978-0226901374

Wilson, D., Kniffin, K. (2003) Altruism from an Evolutionary Perspective. *Research on Altruism and Love: An annotated bibliography of major studies in sociology, evolutionary biology, and theology*, pp. 117-136. S. Post, B. Johnson, M. McCullough, J. Schloss (Eds.), Radnor, PA: Templeton Foundation Press, 2003. Available at SSRN: https://ssrn.com/abstract=2957991
